# Resource implications of the latent tuberculosis cascade of care: a time and motion study in five countries

**DOI:** 10.1186/s12913-020-05220-7

**Published:** 2020-04-21

**Authors:** H. Alsdurf, O. Oxlade, M. Adjobimey, F. Ahmad Khan, M. Bastos, N. Bedingfield, A. Benedetti, D. Boafo, T. N. Buu, L. Chiang, V. Cook, D. Fisher, G. J. Fox, F. Fregonese, P. Hadisoemarto, J. C. Johnston, F. Kassa, R. Long, S. Moayedi Nia, T. A. Nguyen, J. Obeng, C. Paulsen, K. Romanowski, R. Ruslami, K. Schwartzman, H. Sohn, E. Strumpf, A. Trajman, C. Valiquette, L. Yaha, D. Menzies

**Affiliations:** 1 Department of Epidemiology, Biostatistics and Occupational Health, McGill University, Montreal, QC Canada; 2 McGill International TB Centre, McGill University, 5252 Boulevard de Maisonneuve, Room 3D.58, Montreal, QC Canada; 3 Respiratory Epidemiology and Clinical Research Unit (RECRU), McGill University, Montreal, QC Canada; 4 Programme National contre la Tuberculose-Bénin, Centre National Hospitalier Universitaire de Pneumo-Phtisiologie-Cotonou, Cotonou, Benin; 5 Social Medicine Institute, State University of Rio de Janeiro, Rio de Janeiro, Brazil; 6 University of Calgary, Calgary, AB Canada; 7 Chest Clinic, Komfo Anokye Teaching Hospital, Kumasi, Ghana; 8 Woolcock Institute of Medical Research, Hanoi, Vietnam; 9 Provincial Tuberculosis Services, British Columbia Centre for Disease Control, Vancouver, BC Canada; 10 Department of Medicine, University of British Columbia, Vancouver, BC Canada; 11 Division of Respiratory Medicine, University of Calgary, Calgary, AB Canada; 12 The Faculty of Medicine and Health, The University of Sydney Central Clinical School, The University of Sydney, Sydney, NSW Australia; 13 Department of Public Health, Faculty of Medicine, TB-HIV Research Center, Universitas Padjadjaran, Bandung, Indonesia; 14 Department of Medicine, Faculty of Medicine and Dentistry, University of Alberta, Edmonton, AB Canada; 15 Department of Social and Preventive Medicine, Université de Montréal, Montreal, QC Canada; 16 Department of Biomedical Sciences, Division of Pharmacology & Therapy, Faculty of Medicine, Universitas Padjadjaran, Bandung, Indonesia; 17 Department of Epidemiology, John Hopkins Bloomberg School of Public Health, Baltimore, MD USA

**Keywords:** Latent tuberculosis infection, Cascade-of-care, Time and motion study

## Abstract

**Background:**

The End TB Strategy calls for global scale-up of preventive treatment for latent tuberculosis infection (LTBI), but little information is available about the associated human resource requirements. Our study aimed to quantify the healthcare worker (HCW) time needed to perform the tasks associated with each step along the LTBI cascade of care for household contacts of TB patients.

**Methods:**

We conducted a time and motion (TAM) study between January 2018 and March 2019, in which consenting HCWs were observed throughout a typical workday. The precise time spent was recorded in pre-specified categories of work activities for each step along the cascade. A linear mixed model was fit to estimate the time at each step.

**Results:**

A total of 173 HCWs in Benin, Canada, Ghana, Indonesia, and Vietnam participated. The greatest amount of time was spent for the medical evaluation (median: 11 min; IQR: 6–16), while the least time was spent on reading a tuberculin skin test (TST) (median: 4 min; IQR: 2–9). The greatest variability was seen in the time spent for each medical evaluation, while TST placement and reading showed the least variability. The total time required to complete all steps along the LTBI cascade, from identification of household contacts (HHC) through to treatment initiation ranged from 1.8 h per index TB patient in Vietnam to 5.2 h in Ghana.

**Conclusions:**

Our findings suggest that the time requirements are very modest to perform each step in the latent TB cascade of care, but to achieve full identification and management of all household contacts will require additional human resources in many settings.

## Background

Tuberculosis (TB) is the leading cause of death due to an infectious disease, killing more people than HIV/AIDS [1]. It is estimated that 1.7 billion people or one quarter of the world’s population have latent TB infection (LTBI) [1, 2]. Between 5 and 15% of these people will develop active TB disease over the course of their lifetime, with higher rates among certain subgroups, such as persons living with HIV, children under 5 years of age and household contacts (HHC) of persons with pulmonary TB [1]. One of the three pillars of the World Health Organization’s (WHO) End TB Strategy is to provide integrated, patient-centered care, particularly therapy aimed at preventing the development of active TB disease in HHC [3]. The 2018 United Nations’ High-Level Meeting (UNHLM) on Tuberculosis resulted in a declaration calling for the scale-up of evaluation and treatment of LTBI for 20 million adult HHC by 2022 [4].

The LTBI cascade of care is a term for the entire patient journey, from identification of a person at risk for LTBI (for example, HHC of a patient with pulmonary TB), to completion of LTBI treatment. In 2016, we published a systematic review and meta-analysis of the LTBI cascade of care which demonstrated that losses at each step of the cascade resulted in fewer than 20% of eligible contacts completing preventive therapy [5]. The healthcare worker (HCW) time required to provide clinical services for the many steps along this LTBI cascade of care remains largely unknown; yet this information is critical for decisions regarding the provision of health care services and to estimate the personnel needed for scale-up of LTBI testing and therapy.

The objectives of our study were to: quantify the time it takes HCWs to perform the work tasks associated with each step along the LTBI cascade of care for HHC in Canada (a high-income country), and in four low-and middle-income countries (LMIC); and to estimate the human resource needs to provide LTBI care to all HHC of new, confirmed, pulmonary TB patients in each of the participating countries.

## Methods

### Parent study

This time and motion (TAM) study was conducted as part of a larger pragmatic, cluster-randomized trial which took place in 24 health facilities in Benin, Canada, Ghana, Indonesia, and Vietnam. The main objective of the parent trial was to evaluate and strengthen the LTBI cascade of care in these settings. The methods of the parent trial are described in detail elsewhere [6].

### Time and motion (TAM) study

HCWs who worked at least one full day per week delivering TB care at all participating health facilities were eligible for the TAM study. At each participating health facility, purposive sampling was used and we aimed to include a minimum of ten HCWs, with at least three HCWs working in each of three cadres: 1) doctors; 2) nurses; 3) other HCWs (i.e. social workers, health assistants, pharmacists, and community health workers).

For each TAM, a participating HCW was observed continuously throughout a typical workday. The TAM consisted of a designated research staff at each site noting down minute-by-minute each activity that the HCW performed throughout the day, and categorizing each activity based on a pre-specified list. After completion of each discrete activity, the worker was asked to categorize that activity into one of three main types: 1) Direct patient care (i.e. any face-to-face encounter, phone call with a patient or patient education); 2) Other clinical activities (i.e. charting, dictations, reviewing laboratory results or x-rays); and 3) Training or administrative tasks (i.e. supervising trainees, meetings or emails). Time spent on breaks (i.e. restroom, meals or personal phone calls) was recorded on the TAMs but removed from all analyses. A patient encounter began at the moment a patient went into the examination room with the HCW being observed, and ended at the time the patient left the room. For each patient encounter, the time recorded included time spent on initial greetings and introductions, explanation, actually performing the activity, then education and instructions, arranging further follow-up if required, and finally completing all related documentation (e.g. charting, completing forms, or filling registries). After these encounters, the HCWs were asked to categorize each patient into three broad types of medical conditions: 1) LTBI; 2) active or suspected active TB; and 3) non-TB, meaning any other medical condition. LTBI patient encounters were further categorized into six specific activities: 1) Identification of contacts; 2) Placing TST or drawing blood samples for IGRA^1^; 3) Reading TST or IGRA; 4) Conducting medical evaluation (e.g. symptom check, physical exam and chest radiography); 5) Recommending and discussing LTBI treatment; and 6) LTBI treatment follow-up visits.

TAMs were scheduled in advance with each HCW for a typical workday, defined as a day in which the HCW did not have any planned or likely change in their normal schedule (such as leaving early to pick up a child or attending a personal appointment). At the start of the TAM day, the local research staff confirmed with the HCW that it should be a typical workday. If there was an unanticipated event during that day, the TAM was stopped and rescheduled for another time.

### Data collection

Data was collected between January 2018 and March 2019. To ensure standardized measurements, all research staff performing the TAMs received initial and refresher training from one investigator (HA) on how to observe and record HCWs time using standard data collection forms, and properly classify and code each observation. All data was recorded on paper data collection sheets, and then de-identified data was transferred to Excel spreadsheets with pre-specified drop-down menus.

### Analyses

Characteristics of the HCWs who performed at least one LTBI patient encounter were compared to HCWs observed with TAMS that did not perform any LTBI patient encounters using a chi-square test for categorical variables.

Data was analyzed for individual LTBI patient encounters. If the time recorded reflected visits with multiple patients simultaneously or activities spanning multiple cascade steps, these observations were excluded from analysis. The mean and median time in minutes each individual HCW spent on each LTBI patient encounter, at each step in the cascade, was estimated for: 1) the type of setting (Canada versus LMIC) and 2) HCW cadre (i.e. doctors, nurses, other HCWs). A linear mixed model (LMM) was fit for each HCW cadre and type of setting, for all steps in the LTBI cascade of care. LMMs were fit for each step in the LTBI cascade of care (i.e. steps #1–6) in order to estimate the effect of the following covariates on HCWs time: 1) HCW cadre (i.e. doctor, nurse, other HCW); 2) TB-specific job role; 3) type of setting (i.e. Canadian vs. LMIC). Interactions, defined a priori*,* were considered between type of setting and HCW cadre and TB-specific job. Final estimates of total HCWs time required at each step in the LTBI cascade presented in the manuscript were based on statistically significant models. We present time estimates from the linear mixed models for the HCW cadre in each setting that were found to perform the majority of LTBI patient encounters at each step. Data were analyzed using SAS version 9.4 (SAS Institute, Cary, USA).

To estimate the human resource requirements for country wide scale-up of LTBI care for all HHC of newly confirmed pulmonary TB patients, we used the linear mixed models estimates for Canada or LMIC settings, of HCW time to complete work tasks for each step in the LTBI cascade of care. For each country, the estimates of time (in hours) from the linear mixed models (from Canada for high income countries and from LMIC for all other countries) at each step were multiplied by the country-specific average number of household contacts per index TB patient (from unpublished parent study results). For the first step of the identification of all household contacts for one index patient, the time per index case was used rather than the time per contact. The time HCWs spent on medical evaluation and recommending / discussing LTBI preventive therapy was multiplied by the prevalence of TST positive contacts in that setting - assumed to be 50% in LMIC and 28% in Canadian sites, based on a published systematic review [7].

Based on the predominance of HCW cadres we observed performing each of the cascade steps, for our extrapolations we assumed that: 1) nurses would perform the contact identification, TST administration and reading, and the LTBI treatment follow-up visits; 2) doctors would perform the medical evaluations; and 3) both doctors and nurses would recommend /discuss LTBI treatment initiation. We assumed monthly follow-up visits during LTBI therapy - meaning three visits for 4 months of rifampin (4R) in Canada, and five visits for 6 months of isoniazid (6H) in LMIC. Time for follow-up visits was multiplied by the prevalence of TST positive household contacts and number of visits (i.e. three visits in Canada and five in LMIC). Finally, the HCW time to conduct all activities at all steps was summed, by HCW cadre, to provide country-specific estimates for the predicted total health care personnel time for all household contacts of one index patient.

### Ethics

The Research Ethics Board of the Research Institute of the McGill University Health Center approved the study. Verbal consent was obtained from all HCWs to permit research staff to observe their daily work activities. For ethical reasons, research staff conducting the TAM did not enter patient rooms during encounters with observed workers.

### Role of the funding source

This study was supported by the Canadian Institutes of Health Research (Grant #FND331745). This funding agency had no role in study design, interpretation or writing of this report. The corresponding author had full access to all the data and had final responsibility for the decision to submit for publication.

## Results

In total, 184 HCWs were approached to participate in the TAMs, of whom 173 (94%) agreed (85 doctors, 76 nurses, and 12 other HCWs). Of these, 83 were observed to have at least one patient encounter at one or more steps along the LTBI cascade of care; the remaining 90 HCWs were not observed to have any LTBI related patient encounters during the day selected for TAM observation (Table 1). A total of 731 patient encounters were recorded on the TAMs across all sites at all steps along the LTBI cascade of care, including 466 and 265 patient encounters at Canadian and LMIC sites, respectively.

**Table 1:** Characteristics of 173 HCW participating in TAMs for the LTBI cascade of care

	HCW with one or more patient encounters along the steps in the LTBI Cascade of Care	HCW with no patient encounters along the steps in the LTBI Cascade of Care	***p***-value^**1**^
	(*N* = 83)	(*N* = 90)	
**Sex**			
Male	27 (54%)	23 (46%)	0.31
Female	56 (46%)	67 (54%)	
**Role/type of patients**			
**TB specific**	73 (66%)	37 (34%)	<0.01
General (all patients)	10 (16%)	53 (84%)	
**HCW category**			
Doctor	31 (36%)	54 (64%)	0.01
Nurse	45 (59%)	31 (41%)	
Other HCW	7 (58%)	5 (42%)	
**Country**			
Benin	5 (28%)	13 (72%)	<0.01
Canada	48 (92%)	4 (8%)	
Ghana	10 (59%)	7 (41%)	
Indonesia	12 (27%)	33 (73%)	
Vietnam	8 (20%)	33 (80%)	
**Country setting**			
High Income^b^	48 (92%)	4 (8%)	<0.01
LMIC^c^	35 (29%)	86 (71%)	

^1^*p*-values from χ^2^ test for difference in characteristics of HCW performing at least one patient encounter along the LTBI cascade of care compared to HCW participating in TAMs but not performing any tasks along LTBI Cascade

^b^High income country (HIC) =Canada

^c^Low-and middle-income countries (LMIC) =Benin, Ghana, Indonesia, Vietnam

### Healthcare worker time requirements

**Fig. 1:**
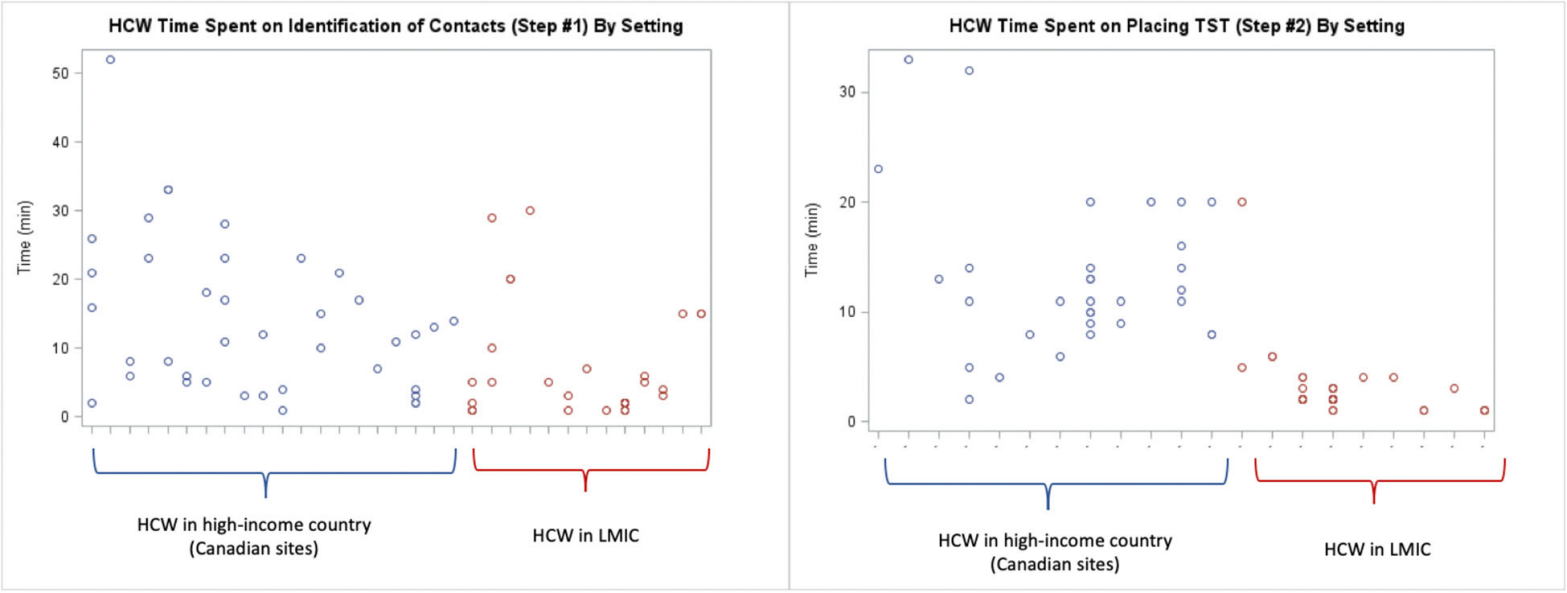
Scatterplot of time for individual HCW-contact encounters: for Identification of Contacts (Step #1) and Placing a TST (Step #2)

**Fig. 2:**
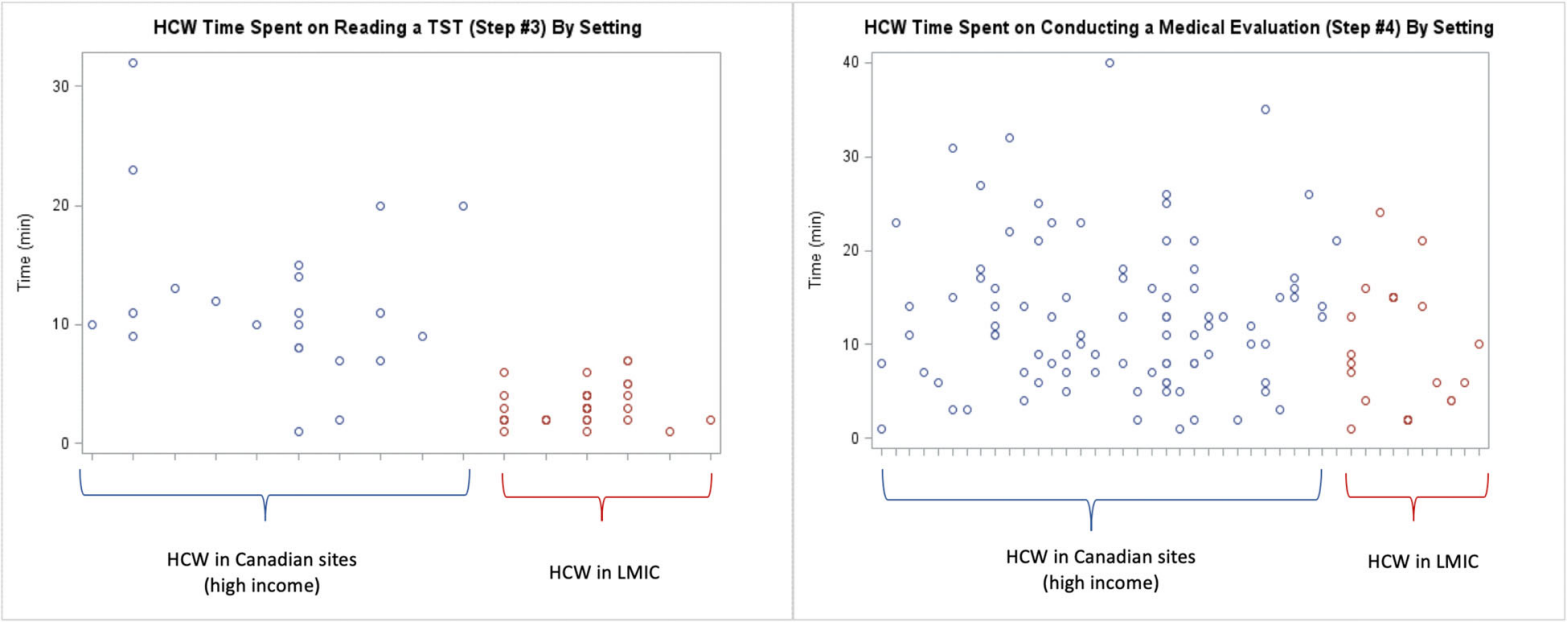
Scatterplot of time for an individual HCW patient encounter for: Reading a TST (Step #3) and Conducting a Medical Evaluation (Step #4)

**Fig. 3:**
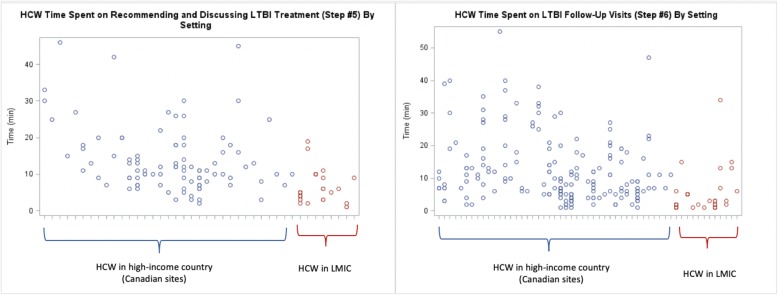
Scatterplot of time for individual HCW-contact encounters: for Recommending LTBI Treatment (Step #5) and LTBI Follow-Up Visit (Step #6). *Note: Each point on the x-axis denotes a separate, individual HCW; each circle is a unique patient encounter (blue = high-income; red = LMIC)

The time to conduct a medical evaluation of a TST positive HHC was the longest, while reading a TST took the least time (Table 2). Placing and reading a TST in LMIC took 2 and 3 min, respectively, compared to 11 and 10 minutes, respectively, in Canada (Table 2). The HCWs time to identify contacts, place and read TSTs, conduct medical evaluations and perform patient follow-up visits was very different in Canada compared to LMIC, as shown in the stratified analysis (Table 2). There was considerable variation in HCWs time to conduct a medical evaluation, but much less variability in the time taken to read a TST (Fig. 2). The variability of the remaining steps is shown in Figs. 1, 2 and 3. Nurses were responsible for most LTBI patient encounters for contact identification, and TST administration and were the only HCW cadre to read TSTs across all sites, while doctors conducted most medical evaluations (Table 3). Both doctors and nurses took part in recommending/discussing LTBI treatment initiation, but nurses performed the majority of follow-up visits (Table 3). The linear mixed models show that the predicted HCW time required for the designated HCWs cadre to perform each step in the LTBI cascade varied significantly by setting (Table 4).

**Table 2:** HCW time^a^ spent on patient encounters at each step along the LTBI Cascade of Care – Canada vs. low- and middle-income countries (Includes initial greeting, explanation, activity, instructions, and documentation)

LTBI Cascade of Care Steps	Number of HCW performing each Step on TAM day	Total number of LTBI patient encounters with HCW at each Step on TAM day	Mean time spent on each Step (Std. Dev.)	Median time spent on each Step (IQR)
**1. Identify contacts (all sites)**	33	73	10.5 (10.4)	6.0 (2, 16)
Canada^a^	20	39	14.0 (11.2)	12.0 (5, 21)
LMIC^b^	13	34	6.6 (8.0)	2.5 (2, 7)
**2. Place TST**^c^** (all sites)**	22	64	8.1 (7.5)	5.5 (2, 12)
Canada	13	32	13.1 (7.1)	11.0 (9, 15)
LMIC	9	32	3.1 (3.4)	2.0 (2, 4)
**3. Read TST**^c^** (all sites)**	17	59	6.4 (6.1)	4.0 (2, 9)
Canada	11	22	11.9 (6.9)	10.5 (8, 14)
LMIC	6	37	3.2 (1.6)	3.0 (2, 4)
**4. Conduct Medical Evaluation (all sites)**	43	116	12.1 (7.8)	11.0 (6, 16)
Canada	33	90	13.0 (7.9)	12.0 (7, 17)
LMIC	10	26	9.0 (6.6)	7.5 (2, 15)
**5. Recommend and discuss LTBI treatment (all sites)**	42	143	10.8 (8.5)	9.0 (4, 13)
Canada	34	92	13.9 (8.9)	11.0 (8, 18)
LMIC	8	51	5.3 (3.5)	4.0 (4, 5)
**6. LTBI treatment follow-up (all sites)**	56	276	9.3 (9.5)	6.0 (2, 12)
Canada	44	191	12.0 (9.9)	9.0 (5, 16)
LMIC	12	85	3.4 (4.4)	2.0 (1, 5)

^a^Time from 83 HCW participating in at least one LTBI patient encounter along the steps of the cascade

^a^Canada is the one high-income country

^b^Low-and middle-income countries (LMIC) include: Benin, Ghana, Indonesia, Vietnam

^c^Steps 2 & 3 may include HCW time spent on patient education, in addition to placing and reading a TST

**Table 3:** HCW time^a^ spent on patient encounters at each step along LTBI Cascade of Care Steps – By HCW cadre

LTBI Cascade of Care Steps	Number of HCW observed performing each Step	Total number of observed HCW-patient encounters at each Step	Mean time (minutes) spent on each encounter (Std. Dev.)	Median time (minutes) spent on each encounter (IQR)
**1. Identify contacts**				
Doctor	16	22	13.0 (7.6)	15.0 (7, 18)
Nurse	16	48	9.2 (11.3)	4.0 (2, 12)
Other HCW^b^	1	3	14.7 (12.7)	10.0 (5, 29)
**2. Place TST**				
Doctor	2	2	4.0 (-)	4.0 (-)
Nurse	19	50	9.7 (7.7)	8.5 (3, 13)
Other HCW^b^	1	12	2.1 (0.7)	2.0 (2, 3)
**3. Read TST**				
Doctor	-	-	-	-
Nurse	17	59	6.4 (6.1)	4.0 (2, 9)
Other HCW^b^	-	-	-	-
**4. Conduct Medical Evaluation**				
Doctor	19	67	12.7 (6.2)	13.0 (8, 16)
Nurse	21	37	11.6 (10.2)	7.0 (3, 16)
Other HCW^b^	3	12	10.3 (7.9)	8.5 (6, 14)
**5. Recommend and discuss LTBI treatment**				
Doctor	20	55	12.3 (7.4)	10.0 (9, 14)
Nurse	20	77	9.7 (9.5)	5.0 (4, 12)
Other HCW^b^	2	11	10.9 (5.2)	11.0 (7, 15)
**6. LTBI treatment follow-up**				
Doctor	17	65	7.2 (5.0)	5.0 (4, 10)
Nurse	34	176	11.0 (9.5)	7.0 (2, 16)
Other HCW^b^	5	35	5.1 (6.6)	2.0 (2, 7)

^a^Time from 83 HCW participating in at least one LTBI patient encounter along the steps of the cascade

^b^Note: Other HCWs include: health assistants, social workers, sociologists and pharmacists

**Table 4:** HCW time required for each encounter/activity in the LTBI Cascade of Care, by country income level and cadre of workers (from linear mixed model)

Model		Time (min)	95% CI*
1. Identify contacts	Nurse in Canada^a^	12.8	(5.1, 20.6)
	Nurse in LMIC^b^	7.5	(0.9, 14.2)
2. Place TST	Nurse in Canada	15.8	(11.4, 20.2)
	Nurse in LMIC	4.5	(0.0*, 9.5)
3. Read TST	Nurse in Canada	12.0	(9.5, 14.5)
	Nurse in LMIC	2.9	(0.0*, 5.8)
4. Conduct medical evaluation	Doctor in Canada	13.1	(10.2, 15.9)
	Doctor in LMIC	9.7	(5.0, 14.4)
1.1.5. Recommend and discuss LTBI treatment^c^	Doctor in Canada	14.3	(9.8, 18.8)
	Nurse in Canada	16.0	(11.3, 20.8)
	Doctor in LMIC	5.5	(0.0*, 11.7)
	Nurse in LMIC	7.2	(1.8, 12.5)
6. LTBI follow-up visit	Nurse in Canada	14.8	(10.3, 19.3)
	Nurse in LMIC	6.5	(1.2, 11.9)

^a^Canada is the one high-income country

^b^LMICs include: Benin, Ghana, Indonesia, and Vietnam

^c^In the LMM for Step #5, there was not a statistically significant interaction between type of HCW and setting, so the expected difference between doctors and nurses is the same whether in HIC or LMIC settings

*Note: Where the CI lower limit is below zero, values were cut-off at 0 minutes

### Human resource requirements

As seen in the country-specific tables, total predicted time (in hours) for each type of HCW to complete all the steps along the LTBI cascade for one index patient ranged from 1.8 h (1.4 nurse hours and 0.4 doctor hours) in Vietnam to 5.2 h (4.1 nurse hours and 1.1 doctor hours) in Ghana (Table 5 and supplemental Tables S1–S5).

**Table 5:** Total time^a^ required for contact management and LTBI care for all steps along the LTBI Cascade of Care

	Identify contacts	Place TST^b^	Read TST^b^	Conduct Medical Evaluation^b,c^	Recommend and Discuss LTBI Treatment^b,c^	LTBI Follow-up Visit^b,c,d^	Total Time Per Index Case (hours)
**Country**	(A)	(B)	(C)	(D)	(E)	(F)	Σ (A-F)
**LMIC**							
Benin							
Doctors	-	-	-	18.9	10.7	-	**0.50**
Nurses	7.5	17.6	11.3	-	14.0	63.4	**1.90**
Ghana							
Doctors	-	-	-	42.7	24.2	-	**1.12**
Nurses	7.5	39.6	25.5	-	31.7	143.0	**4.12**
Indonesia							
Doctors	-	-	-	16.0	9.1	-	**0.42**
Nurses	7.5	14.9	9.6	-	11.9	53.6	**1.63**
Vietnam							
Doctors	-	-	-	13.6	7.7	-	**0.36**
Nurses	7.5	12.6	8.1	-	10.1	45.5	**1.40**
**High-Income**							
Canada							
Doctors	-	-	-	13.1	14.3	-	**0.46**
Nurses	12.8	56.9	43.2	-	16.0	44.4	**2.89**

^a^Predicted time (min) for each step from linear mixed models (LMM) shown in Supplementary Tables S1–5

^b^Assumes step accounts for all household contacts (HHC) for one index case; based on average number of HHC per index patient in each country observed in the main study: Benin = 3.9; Ghana = 8.8; Indonesia = 3.3; Vietnam = 2.8; Canada = 3.6

^c^Assumes a prevalence of TST positive for HHC of index patient is 50% in LMIC and 28% in Canada (Fox 2013)

^d^Assumes 5 follow-up visits for 6 months of INH treatment of LTBI in LMIC and 3 follow-up visits for 4 months of RIF treatment of LTBI in Canada

## Discussion

This study provides important information on the staffing resources needed to ensure that all household contacts of new, pulmonary TB patients are provided with high quality patient-centered care, a focus of the End TB strategy [3]. This study provides estimates of the amount of time taken by different cadres of HCW in very different settings on specific activities required at all the steps in the LTBI cascade, using a method developed to precisely measure time on specific work tasks [8]. Although the overall estimated human resources required for direct LTBI related patient care appears modest in most settings, in the LMIC included in this study, there are fewer than four doctors and 12 nurses per 10,000 population [9]. Hence, even a modest increase in number of HCWs would be an important undertaking for local health systems.

Our study captured systematic differences in HCW time needed to conduct patient care activities at each step in the LTBI cascade of care between Canadian sites and LMIC. TB clinics and health facilities in LMIC had much higher number of patient visits per HCW per day, on average, therefore HCW had less time to dedicate to each patient encounter. The greater resources in Canadian sites allowed for longer patient encounters. This enabled more patient education and counselling as routine components of LTBI care. This highlights the need for additional human resources in LMIC in order to ensure comprehensive, quality LTBI related patient care.

There were a number of important limitations to our study. The TAMs relied upon HCWs to perform activities associated with the steps in the cascade during the selected TAM days in order to gather information on the time required for each step. However, many of the HCWs who participated in TAMs did not have LTBI-related patient encounters on the day of observation. Our predicted estimates of HCWs time are not based on the actual trajectory of individual household contacts through all steps of the LTBI cascade of care but are ‘reconstructed’ based on separate patient encounters for each cascade step. Following a single household contact would have required multiple TAM days specifically tracking each contact, which would have been impractical.

Since HCWs are being shadowed by an observer recording their every activity throughout their entire workday, it is impossible to eliminate the potential for the Hawthorne effect. While being observed on the TAM day, it is plausible that HCWs took fewer breaks and may have spent more time with each patient encounter. However, all break time was removed in the analysis, and it seems unlikely there would have been a differential increase in HCWs time with one type of patient, or one particular activity, rather they may have increased slightly their time on all patient visits and activities. For ethical reasons, the research staff did not directly observe patient encounters, but only recorded the time the encounter started with initial greetings and ended with completion of documentation. Hence there may have been time spent on chatting about unrelated things (the weather, or Donald Trump), but this reflects the reality of human encounters, and so provides a more realistic estimate of the true time needed. The observers also relied on what the healthcare worker stated was the activity and type of patient, which may have led to some misclassification, although systematic misclassification seems implausible.

The estimates of HCWs resource needs for full contact investigation per index TB patient assumed a very efficient process and so may underestimate the human resource requirements. For example, two HCWs may perform the same task for one contact, or multiple patient visits may be required to complete the same step, such as medical evaluation. HCWs time on each step was based solely on observed patient encounters; other related activities such as checking lab results later, were not counted. Yet the time for direct patient care must be supported by time for other clinical activities such as correspondence and consultations or reviewing investigations. Administrative, training and other non-patient care related activities also account for some part of clinical healthcare personnel time, but these activities were also not included. Hence, we may have underestimated the total personnel time requirements. We included the time required for treatment follow-up visits in our estimates; however, if treatment regimens were shorter (e.g. 4R), then required personnel time would be less.

The health facilities that participated in the parent study may not be generalizable to all health facilities in each country, or to all LMIC, since not all LMIC have similar LTBI practices. Patient and health system differences between facilities, such as greater or lesser need to use translation services for patient encounters in high-income countries (i.e. Canada) may lead to variation from the HCWs time measured in this study. A clinic-based healthcare service delivery model was used for this study which may not be generalizable to other settings with community-based healthcare delivery.

Nevertheless, this study had a number of strengths, particularly that the TAMs captured data on many patient encounters at each step. For example, we observed 143 HCW-contact encounters for recommending LTBI treatment, and 276 LTBI follow-up visits. Selection bias should have been minimal as more than 94% of HCWs participated, and the characteristics of HCWs performing none, or at least one LTBI related activity, were similar. We counted the full time required for each patient encounter, from initial introduction to completion of documentation, and the patient encounters were part of workers’ normal tasks on a routine day, ensuring a realistic estimation of the time required. Prior cost-effectiveness analyses (CEA) [10–17] have used time estimates from third party payment schemes [18–20] to calculate HCWs time required to perform an activity, like placing TST, and associated costs. However, we directly measured the time and estimated the variability of time by setting and cadre; these estimates should be useful to inform future costing studies as well as health administrators’ planning new LTBI programmes or scale-up of LTBI services. Numerous TAM studies have been conducted on time allocation of HCW [21–25], but no study focused specifically on LTBI related activities. Thus, our study contributes important information about HCW time requirements to perform LTBI related patient care activities.

Another strength of our study is that time was estimated for each step in the LTBI cascade for Canada and LMIC separately, in order to provide setting-specific estimates. Other studies have outlined the treatment phase costs for each HHC to complete preventive therapy [15, 26] but our study includes the HCWs time for pre-treatment phase encounters – which accounted for more personnel time than treatment follow-up in this study.

WHO recommends scaling-up LTBI services for HHC, including all persons over 5 years of age [27]; this is likely to dramatically increase the numbers of people accessing LTBI services globally, particularly in high burden LMIC [1]. Our study demonstrates that additional healthcare workers will be needed in the workforce to ensure adequate human resources to identify, screen and treat all close contacts. Our study also demonstrates that tuberculin skin testing and reading in the LMIC settings observed required very little time, which is an important consideration in terms of the implementation of LTBI testing as part of routine management of non-HIV infected household contacts. This study provides TB programs with the tools to calculate the additional personnel needed to perform all the steps of the LTBI cascade based on the number of active, pulmonary TB patients in their setting. These estimates could be used to benchmark efficient delivery of LTBI treatment, by determining the number of additional personnel that would need to be hired and trained for LTBI program scale-up.

## Conclusion

The UNHLM recognized the need for increased healthcare services in order to effectively decrease the reservoir of LTBI [4]. If we, the global TB community, are serious about decreasing the reservoir of LTBI, we must address the human resource needs – the time and energy – it will take well-trained healthcare professionals to do this work. Strong political and financial commitments will be needed from national TB programs to support the expansion of LTBI services in order to provide high quality patient-centered care at all steps in the LTBI cascade of care.

## Supplementary information

**Additional file 1: Supplemental Table 1.** Predicted health care personnel time to perform all tasks in the LTBI Cascade of Care for all household contacts (HHC) of one index patient in Benin (observed data: 3.9 HHC per index patient). **Supplemental Table 2.** Predicted health care personnel time required for HCW to perform all tasks in the LTBI Cascade of Care for all household contacts (HHC) of one index patient in Canada (observed data: 3.6 HHC per index patient). **Supplemental Table 3.** Predicted health care personnel time required for HCW to perform all tasks in the LTBI Cascade of Care for all household contacts (HHC) of one index patient in Ghana (observed data: 8.8 HHC per index patient). **Supplemental Table 4.** Predicted health care personnel time required for HCW to perform all tasks in the LTBI Cascade of Care for all household contacts (HHC) of one index patient in Indonesia (observed data: 3.3 HHC per index patient). **Supplemental Table 5.** Predicted health care personnel time required for HCW to perform all tasks in the LTBI Cascade of Care for all household contacts (HHC) of one index patient in Vietnam (observed data: 2.8 HHC per index patient).

## Data Availability

Aggregate data at the country level will be available once the study has been published and secondary analyses are complete, upon written request and provision of a detailed statistical analysis plan to the authors.

## Abbreviations

HCW: Healthcare workers
HHC: Household contacts
IGRA: Interferon-gamma release assay
LLM: Linear mixed model
LTBI: Latent tuberculosis infection
LMIC: Low- and middle-income countries
TAM: Time and motion studies
TB: Tuberculosis
TST: Tuberculin skin test
UNHLM: United Nations High-Level Meeting
WHO: World Health Organization

## Footnotes

1 There were only 13 observations noted specifically to be IGRA blood draws at Canadian sites
