# Supplementary material for: Resource implications of the latent tuberculosis cascade of care: a time and motion study in five countries
Source: BMC Health Serv Res. 2020 Apr 21;20:341. doi: 10.1186/s12913-020-05220-7 (PMC7175545; doi:10.1186/s12913-020-05220-7)
Supplement: Supplementary file 1 — Additional file 1: Supplemental Table 1. Predicted health care personnel time to perform all tasks in the LTBI Cascade of Care for all household contacts (HHC) of one index patient in Benin (observed data: 3.9 HHC per index patient). Supplemental Table 2. Predicted health care personnel time required for HCW to perform all tasks in the LTBI Cascade of Care for all household contacts (HHC) of one index patient in Canada (observed data: 3.6 HHC per index patient). Supplemental Table 3. Predicted health care personnel time required for HCW to perform all tasks in the LTBI Cascade of Care for all household contacts (HHC) of one index patient in Ghana (observed data: 8.8 HHC per index patient). Supplemental Table 4. Predicted health care personnel time required for HCW to perform all tasks in the LTBI Cascade of Care for all household contacts (HHC) of one index patient in Indonesia (observed data: 3.3 HHC per index patient). Supplemental Table 5. Predicted health care personnel time required for HCW to perform all tasks in the LTBI Cascade of Care for all household contacts (HHC) of one index patient in Vietnam (observed data: 2.8 HHC per index patient). [file 12913_2020_5220_MOESM1_ESM.docx]

**Resource Implications of the latent tuberculosis cascade of care: Supplemental Tables**

**Supplemental Table 1:** Predicted health care personnel time to perform all tasks in the LTBI Cascade of Care for all household contacts (HHC) of one index patient in **Benin** (observed data: 3.9 HHC per index patient)

|  | **Predicted time, in minutes, for each cadre of HCW to perform each type of patient care encounter^1^** | **Predicted time, in hours, for each cadre of HCW to perform each patient care encounter** | **Multiplier^2^** | **Predicted total time, in hours, for HCW to perform each step for all contacts of one index patient** |
| --- | --- | --- | --- | --- |
| **LTBI Cascade of Care Steps**  (type of HCW performing step) | (A) | (B) | (E) | (F) = (D) x (E) |
| **1. Identify contacts^3^** |  |  |  |  |
| Nurse | 7.5 | 0.13 | 1.0 | 0.13 |
| **2. Place TST** |  |  |  |  |
| Nurse | 4.5 | 0.08 | 3.9 | 0.29 |
| **3. Read TST** |  |  |  |  |
| Nurse | 2.9 | 0.05 | 3.9 | 0.19 |
| **4. Conduct Medical Evaluation** |  |  |  |  |
| Doctor | 9.7 | 0.16 | 1.95 | 0.32 |
| **5. Recommend and Discuss LTBI Treatment** |  |  |  |  |
| Doctor | 5.5 | 0.09 | 1.95 | 0.18 |
| Nurse | 7.2 | 0.12 | 1.95 | 0.23 |
| **6. LTBI Follow-up Visit** |  |  |  |  |
| Nurse | 6.5 | 0.11 | 9.75 | 1.06 |
| **Total time to complete all steps** - All personnel |  |  |  | **2.40** |
| **Total time for a Doctor** |  |  |  | **0.50** |
| **Total time for a Nurse** |  |  |  | **1.90** |

^1^Predicted time (min) for each step from linear mixed models (LMM) is the average for all LMICs shown in Table 4. ^2^The multiplier at steps #2-5 is 3.9 which was the average number of household contacts (HHC) per index patient in Benin observed in the main study; steps #4-5 are also multiplied by 0.50, assuming a prevalence of 50% TST positive among all HHC of index patients (Fox 2013); step #6 is multiplied by 9.75, based on an assumed 5 follow-up visits (5 x 1.95= 9.75) for 6 months INH treatment of LTBI in 1.95 HHC per inex case. ^3^The time on this step accounts for identification of all HHC of one index patient.

**Supplemental Table 2:** Predicted health care personnel time required for HCW to perform all tasks in the LTBI Cascade of Care for all household contacts (HHC) of one index patient in **Canada** (observed data: 3.6 HHC per index patient)

|  | **Predicted time, in minutes, for each cadre of HCW to perform each type of patient care encounter^1^** | **Predicted time, in hours, for each cadre of HCW to perform each patient care encounter** | **Multiplier^2^** | **Predicted total time, in hours, for HCW to perform each step for all contacts of one index patient** |
| --- | --- | --- | --- | --- |
| **LTBI Cascade of Care Steps**  (type of HCW performing step) | (A) | (B) | (E) | (F) = (D) x (E) |
| **1. Identify contacts^3^** |  |  |  |  |
| Nurse | 12.8 | 0.21 | 1.0 | 0.21 |
| **2. Place TST** |  |  |  |  |
| Nurse | 15.8 | 0.26 | 3.6 | 0.95 |
| **3. Read TST** |  |  |  |  |
| Nurse | 12.0 | 0.20 | 3.6 | 0.72 |
| **4. Conduct Medical Evaluation** |  |  |  |  |
| Doctor | 13.1 | 0.22 | 1.0 | 0.22 |
| **5. Recommend and Discuss LTBI Treatment** |  |  |  |  |
| Doctor | 14.3 | 0.24 | 1.0 | 0.24 |
| Nurse | 16.0 | 0.27 |  | 0.27 |
| **6. LTBI Follow-up Visit** |  |  |  |  |
| Nurse | 14.8 | 0.25 | 3.0 | 0.74 |
| **Total time to complete all steps** - All personnel |  |  |  | **3.35** |
| **Total time for a Doctor** |  |  |  | **0.46** |
| **Total time for a Nurse** |  |  |  | **2.89** |

^1^Predicted time (min) for each step from linear mixed models (LMM) is the average for all Canadian sites shown in Table 4. ^2^The multiplier at steps #2-5 is 3.6 which was the average number of household contacts (HHC) per index patient in Canada observed in the main study; steps #4-5 are also multiplied by 0.28, assuming a prevalence of 28% TST positive among all HHC of index patients (Fox 2013); step #6 is multiplied by 3.0, based on an assumed 3 follow-up visits (3 x 1.0 = 3.0) for 4 months of RIF treatment of LTBI in 1.0 HHC per index case. ^3^The time on this step accounts for identification of all HHC of one index patient.

**Supplemental Table 3:** Predicted health care personnel time required for HCW to perform all tasks in the LTBI Cascade of Care for all household contacts (HHC) of one index patient in **Ghana** (observed data: 8.8 HHC per index patient)

|  | **Predicted time, in minutes, for each cadre of HCW to perform each type of patient care encounter^1^** | **Predicted time, in hours, for each cadre of HCW to perform each patient care encounter** | **Multiplier^2^** | **Predicted total time, in hours, for HCW to perform each step for all contacts of one index patient** |
| --- | --- | --- | --- | --- |
| **LTBI Cascade of Care Steps**  (type of HCW performing step) | (A) | (B) | (E) | (F) = (D) x (E) |
| **1. Identify contacts^3^** |  |  |  |  |
| Nurse | 7.5 | 0.13 | 1.0 | 0.13 |
| **2. Place TST** |  |  |  |  |
| Nurse | 4.5 | 0.08 | 8.8 | 0.66 |
| **3. Read TST** |  |  |  |  |
| Nurse | 2.9 | 0.05 | 8.8 | 0.43 |
| **4. Conduct Medical Evaluation** |  |  |  |  |
| Doctor | 9.7 | 0.16 | 4.4 | 0.71 |
| **5. Recommend and Discuss LTBI Treatment** |  |  |  |  |
| Doctor | 5.5 | 0.09 | 4.4 | 0.40 |
| Nurse | 7.2 | 0.12 | 4.4 | 0.53 |
| **6. LTBI Follow-up Visit** |  |  |  |  |
| Nurse | 6.5 | 0.11 | 22.0 | 2.38 |
| **Total time to complete all steps** - All personnel |  |  |  | **5.24** |
| **Total time for a Doctor** |  |  |  | **1.11** |
| **Total time for a Nurse** |  |  |  | **4.13** |

^1^Predicted time (min) for each step from linear mixed models (LMM) is the average for all LMICs shown in Table 4. ^2^The multiplier at steps #2-5 is 8.8 which was the average number of household contacts (HHC) per index patient in Ghana observed in the main study; steps #4-5 are also multiplied by 0.50, assuming a prevalence of 50% TST positive among all HHC of index patients (Fox 2013); step #6 is multiplied by 22.0, based on an assumed 5 follow-up visits (5 x 4.4= 22.0) for 6 months INH treatment of LTBI in 4.4 HHC per index case. ^3^The time on this step accounts for identification of all HHC of one index patient.

**Supplemental Table 4:** Predicted health care personnel time required for HCW to perform all tasks in the LTBI Cascade of Care for all household contacts (HHC) of one index patient in **Indonesia** (observed data: 3.3 HHC per index patient)

|  | **Predicted time, in minutes, for each cadre of HCW to perform each type of patient care encounter^1^** | **Predicted time, in hours, for each cadre of HCW to perform each patient care encounter** | **Multiplier^2^** | **Predicted total time, in hours, for HCW to perform each step for all contacts of one index patient** |
| --- | --- | --- | --- | --- |
| **LTBI Cascade of Care Steps**  (type of HCW performing step) | (A) | (B) | (E) | (F) = (D) x (E) |
| **1. Identify contacts^3^** |  |  |  |  |
| Nurse | 7.5 | 0.13 | 1.0 | 0.13 |
| **2. Place TST** |  |  |  |  |
| Nurse | 4.5 | 0.08 | 3.3 | 0.25 |
| **3. Read TST** |  |  |  |  |
| Nurse | 2.9 | 0.05 | 3.3 | 0.16 |
| **4. Conduct Medical Evaluation** |  |  |  |  |
| Doctor | 9.7 | 0.16 | 1.65 | 0.27 |
| **5. Recommend and Discuss LTBI Treatment** |  |  |  |  |
| Doctor | 5.5 | 0.09 | 1.65 | 0.15 |
| Nurse | 7.2 | 0.12 |  | 0.20 |
| **6. LTBI Follow-up Visit** |  |  |  |  |
| Nurse | 6.5 | 0.11 | 8.25 | 0.89 |
| **Total time to complete all steps** - All personnel |  |  |  | **2.05** |
| **Total time for a Doctor** |  |  |  | **0.42** |
| **Total time for a Nurse** |  |  |  | **1.63** |

^1^Predicted time (min) for each step from linear mixed models (LMM) is the average for all LMICs shown in Table 4. ^2^The multiplier at steps #2-5 is 3.3 which was the average number of household contacts (HHC) per index patient in Indonesia observed in the main study; steps #4-5 are also multiplied by 0.50, assuming a prevalence of 50% TST positive among all HHC of index patients (Fox 2013); step #6 is multiplied by 8.25, based on an assumed 5 follow-up visits (5 x 1.65=8.25) for 6 months INH treatment of LTBI in 1.65 HHC per index case. ^3^The time on this step accounts for identification of all HHC of one index patient.

**Supplemental Table 5:** Predicted health care personnel time required for HCW to perform all tasks in the LTBI Cascade of Care for all household contacts (HHC) of one index patient in **Vietnam** (observed data: 2.8 HHC per index patient)

|  | **Predicted time, in minutes, for each cadre of HCW to perform each type of patient care encounter^1^** | **Predicted time, in hours, for each cadre of HCW to perform each patient care encounter** | **Multiplier^2^** | **Predicted total time, in hours, for HCW to perform each step for all contacts of one index patient** |
| --- | --- | --- | --- | --- |
| **LTBI Cascade of Care Steps**  (type of HCW performing step) | (A) | (B) | (E) | (F) = (D) x (E) |
| **1. Identify contacts^3^** |  |  |  |  |
| Nurse | 7.5 | 0.13 | 1.0 | 0.13 |
| **2. Place TST** |  |  |  |  |
| Nurse | 4.5 | 0.08 | 2.8 | 0.21 |
| **3. Read TST** |  |  |  |  |
| Nurse | 2.9 | 0.05 | 2.8 | 0.14 |
| **4. Conduct Medical Evaluation** |  |  |  |  |
| Doctor | 9.7 | 0.16 | 1.4 | 0.23 |
| **5. Recommend and Discuss LTBI Treatment** |  |  |  |  |
| Doctor | 5.5 | 0.09 | 1.4 | 0.13 |
| Nurse | 7.2 | 0.12 |  | 0.17 |
| **6. LTBI Follow-up Visit** |  |  |  |  |
| Nurse | 6.5 | 0.11 | 7.0 | 0.76 |
| **Total time to complete all steps** - All personnel |  |  |  | **1.77** |
| **Total time for a Doctor** |  |  |  | **0.36** |
| **Total time for a Nurse** |  |  |  | **1.41** |

^1^Predicted time (min) for each step from linear mixed models (LMM) is the average for all LMICs shown in Table 4. ^2^The multiplier at steps #2-5 is 2.8 which was the average number of household contacts (HHC) per index patient in Vietnam observed in the main study; steps #4-5 are also multiplied by 0.50, assuming a prevalence of 50% TST positive among all HHC of index patients (Fox 2013); step #6 is multiplied by 7.0, based on an assume 5 follow-up visits (5 x 1.4= 7.0) for 6 months INH treatment of LTBI in 1.4 HHC per index case. ^3^The time on this step accounts for identification of all HHC of one index patient.
